# 自噬在眼镜蛇神经毒素诱导A549细胞死亡中的作用

**DOI:** 10.3779/j.issn.1009-3419.2013.07.02

**Published:** 2013-07-20

**Authors:** 健 申, 靖康 何, 兴 唐, 蓉 韩, 睿 李, 澄澄 徐, 亚娟 吴

**Affiliations:** 1 215000 苏州，苏州大学附属第一医院 The First Affiliated Hospital of Suzhou University, Suzhou 215000, China; 2 215123 苏州，苏州大学药学院 School of Pharmaceutical Sciences, Soochow University, Suzhou 215123, China

**Keywords:** 眼镜蛇神经毒素, 人肺A549腺癌细胞, 自噬, P38-MAPK, Cobrotoxin, Anti-tumor, Autophagy, P38-MARK

## Abstract

**背景与目的:**

已有的研究表明眼镜蛇神经毒素具有抗肿瘤作用，而其在肺癌中的作用罕有研究。本研究观察cobrotoxin对人肺A549腺癌细胞株的抗肿瘤作用，探讨自噬和P38-MAPK通路在这过程中的作用。

**方法:**

应用MTT法观察cobrotoxin对A549细胞和HFL1人肺成纤维细胞的生长抑制作用以及用3-MA抑制自噬、SB203580抑制P38-MAPK通路后cobrotoxin对A549细胞的生长抑制作用; 应用细胞集落平板克隆实验检测cobrotoxin对A549细胞的集落形成的影响; 蛋白免疫印迹法（Western blot）测定单独cobrotoxin作用、cobrotoxin分别联合3-MA抑制自噬和SB203580抑制P38-MAPK通路活性后A549细胞内beclin-1、LC3、P62、P38及pP38等蛋白表达水平。

**结果:**

不同浓度cobrotoxin对A549细胞的生长有明显抑制作用，对HFL1人肺成纤维细胞无明显抑制作用，3-MA、SB203580作用后cobrotoxin对A549细胞的抑制作用降低; 不同浓度cobrotoxin可明显抑制A549细胞的集落形成; cobrotoxin作用后beclin-1、pP38蛋白表达水平明显提高，P62表达明显降低，Ⅱ型/Ⅰ型LC3比值增大，并有浓度依赖性，3-MA抑制自噬后beclin-1蛋白表达水平降低，P62表达增高、Ⅱ型/Ⅰ型LC3比值减小，SB203580抑制P38-MAPK通路后beclin-1、pP38蛋白表达水平降低，Ⅱ型/Ⅰ型LC3比值减小。

**结论:**

cobrotoxin对人肺A549腺癌细胞体外生长有抑制作用，可能通过活化P38-MAPK通路激活自噬参与抗肿瘤过程。

## Abstract (en)

**Background and objective:**

It has been proven that cobrotoxin has anti-tumor effect while its role in lung cancer is rarely studied. The aim of this study is to assay the anti-tumor effect of cobrotoxin in cell line A549, and also to explore its possible mechanism related to autophagy and P38-MARK pathway.

**Methods:**

Using MTT method to observe the inhibition effect of cobrotoxin on the growth of adenocarcinoma cell A549 and human lung fibroblast cell HFL1, as well as on that of A549 pretreated with 3-MA and SB203580, which are the inhibitor of autophagy and P38-MARK pathway respectively. Cell colony tablet cloning experiment was executed to detect the effect of cobrotoxin on colony formation of A549. Determining the protein levels of beclin-1, LC3, p38 and pP38 in A549 by Western blot after cells were exposed to cobrotoxin, or to cobrotoxin combined with either 3-MA or SB203580.

**Results:**

All of different concentrations of cobrotoxin inhibited the growth of A549, but had no obvious effect on that of HFL1. After treating with 3-MA or SB203580, the suppress effect of cobrotoxin on A549 reduced. What's more, different concentrations of cobrotoxin all significantly suppressed the colony formation of A549. The expression of beclin-1 and pP38 in A549 increased obviously after exposure to cobrotoxin, and also the ratio of LC3 Ⅱ to LC3 Ⅰ amplified with a dose-dependant manner, but P62 decreased. The protein level of beclin-1 and the ratio of LC3 Ⅱ to LC3 Ⅰ in cells pretreated with 3-MA were reduced, while that of p62 was increased. Also, in cells that treated with SB203580 before exposed to cobrotoxin, the expression of beclin-1, pP38 and the ratio of of LC3 Ⅱ to LC3 Ⅰ were reduced, but the expression of P62 increased.

**Conclusion:**

Cobrotoxin can suppress the growth of A549 *in vitro*. And the activating of P38-MARK pathway, then upregulating autophagy, was involved in cobrotoxin induced anti-tumor process.

非小细胞肺癌（non-small cell lung cancer, NSCLC）是目前常见的恶性肿瘤之一，对于其治疗，主要是以手术为主的综合治疗，但疗效不尽如人意，对于NSCLC抗癌药物的研发、已投入了大量的人力、物力，取得了许多重要的成就，其中之一就是从天然物质中寻找抗肿瘤活性成分^[1, 2]^。cobrotoxin是从眼镜蛇的毒素中分离出来的一种神经毒素，近年来有国内外文献报道cobrotoxin具有抗肿瘤作用^[3-5]^。本实验研究中，我们以人肺A549腺癌细胞株为实验对象，观察cobrotoxin的抗肿瘤作用并探讨其可能的分子机制，为今后cobrotoxin应用于临床提供理论依据。

## 1 材料与方法

### 1.1 细胞培养

人肺A549腺癌细胞株、人肺成纤维细胞株（购自上海生科院），均为贴壁生长，A549腺癌细胞培养于含10%胎牛血清的PRMI-1640培养液中，人肺成纤维细胞培养于含10%小牛血清的DMEM培养基中，均在5%CO2的37 oC培养箱中培养，待长至80%-90%融合时以0.25%胰酶消化传代，取对数生长期细胞用于实验。

### 1.2

药物、主要试剂、仪器cobrotoxin（由苏州大学医学部药学院药理学系馈赠），3-甲基腺嘌呤（3-MA，美国Sigma公司），SB203580（碧云天公司），二甲基亚砜（DMSO），四甲基偶氮唑盐（MTT，美国Sigma公司），自动酶标仪（Benchmark，美国BID-RAD公司），二氧化碳孵育箱（Hera cell150，德国Heraeus），SDS-PAGE电泳仪，电转移槽。

### 1.3 细胞生长抑制实验（MTT法）

取对数生长期的A549细胞和HFL1细胞，经0.25%胰酶消化后制成含细胞1.0×10^5^/mL的单细胞悬液，接种于96孔培养板中，每孔100 μL，每组设6个平行孔，24 h细胞贴壁后实验组换用含5 μg/mL cobrotoxin、10 μg/mL cobrotoxin、20 μg/mL cobrotoxin、10 μg/mL cobrotoxin+5 μmmol/L 3-MA、10 μg/mL cobrotoxin+10 μmmol/L SB203580、5 μmmol/L 3-MA、10 μmmol/L SB203580不同培养液，设阴性对照组和空白调零孔。培养48 h每孔加MTT（5 mg/mL）20 μL，于孵箱继续作用4 h，弃上清每孔加DMSO 150 μL，微振10 min，用酶标仪测定570 nm附近光吸收值（OD值），实验重复3次，结果取平均值。细胞增殖抑制率（%）=（1-实验组平均OD值/对照组平均OD值）×100%。

### 1.4 细胞集落平板克隆实验

取对数生长期的A549细胞，经0.25%胰酶消化后制成单细胞悬液（400 cell/mL），接种于24孔板，每孔200 cell。接种后于培养箱静置24 h后，每孔换用含5 μg/mL、10 μg/mL、20 μg/mL三个不同浓度cobrotoxin的培养液，设阴性对照组，每组设6个平行孔，作用48 h后各实验组更换为PRMI-1640完全培养基，每隔2天-3天更换培养液1次，培养2周后用甲醇固定，Giemsa工作液染色，于显微镜下计数50个细胞以上的集落数。集落形成抑制率（%）=（对照组集落形成率-实验组集落形成率）/对照组集落形成率×100%。

### 1.5 蛋白免疫印迹法（Western blot）测定beclin-1、LC3、P62、P38及pP38蛋白的表达

0.25%胰酶消化对数生长期的A549贴壁细胞，制备成单细胞悬液，调整细胞密度为1.0×10^5^/mL，分别接种于6孔培养板中，实验组A549细胞分别加入含5 μg/mL cobrotoxin、10 μg/mL cobrotoxin、20 μg/mL cobrotoxin、10 μg/mL cobrotoxin+5 μmmol/L 3-MA、10 μg/mL cobrotoxin+10 μmmol/L SB203580的培养液，阴性对照组A549细胞加入不含cobrotoxin的PRMI-1640完全培养液，作用48 h后收集细胞，加入50 μL蛋白裂解液，混匀后移至EP管中置于冰上超声30 min，然后在4 oC下12, 000 r/min离心5 min，取上清，BCA法测定蛋白浓度，上样前蛋白煮沸变性，于SDS-PAGE凝胶中电泳分离，转膜（硝酸纤维素膜PVDF）免疫杂交，一抗为兔抗人beclin-1单克隆抗体，兔抗人LC3单克隆抗体，兔抗人pP38单克隆抗体，兔抗人P38单克隆抗体，以β-actin为内参，二抗为相应的HRP标记的抗体。化学发光检测法测定蛋白表达结果。

### 1.6 统计学方法

计量资料用Mean±SD表示，实验组与对照组采用两样本均数*t*检验，组间比较采用SPSS 16.0统计软件进行单因素方差分析，检验水准α=0.05，*P* < 0.05为差异有统计学意义。

## 2 结果

### 2.1 Cobrotoxin对A549细胞和HFL1细胞生长的抑制作用

作用48 h后不同浓度cobrotoxin对A549细胞的生长均有明显抑制作用，MTT法测得OD值与对照组相比差异有统计学意义（*P* < 0.05），不同浓度的抑制率分别为21.8%（cobrotoxin 5 μg/mL）、34.2%（cobrotoxin 10 μg/mL）、53.0%（cobrotoxin 20 μg/mL）（*P* < 0.05）。不同浓度的细胞生长抑制率见表 1。不同浓度cobrotoxin对HFL1细胞的生长无明显影响。

**1:** cobrotoxin对A549细胞生长的影响 The effect of cobrotoxin on the growth of cell A549

Cobrotoxin concentration	OD value	*P* (contrast with cobrotoxin group)	Inhibition rate
Control group	0.732±0.091		
5 μg/mL	0.572±0.114	0.027	21.80%
10 μg/mL	0.482±0.035	0.013	34.20%
20 μg/mL	0.344±0.021	0.004	53.00%

### 2.2 3-MA、SB203580干预后cobrotoxin对A549细胞的生长抑制作用

作用48 h后cobrotoxin+3-MA、cobrotoxin+SB203580干预组对A549细胞的生长仍有明显抑制作用，抑制率分别为27.0%（cobrotoxin+3-MA）、24.5%（cobrotoxin+SB203580）但较cobrotoxin组（10 μg/mL）抑制作用变弱，差异有统计学意义（*P* < 0.05）; 3-MA和SB203580单独应用组对A549细胞的生长无明显抑制作用（*P* > 0.05）（表 2）。

**2:** 3-MA、SB203580干预后cobrotoxin对A549细胞生长的影响 The effect of cobrotoxin on the growth of cell A549 after the intervention of 3-MA, SB203580

Group	OD value	*P* (contrast with cobrotoxin group)	Inhibition rate
Control group	0.732±0.091		
Cobrotoxin (10 μg/mL)	0.482±0.035		34.20%
Cobrotoxin+3-MA	0.534±0.121	0.03	27.00%
Cobrotoxin+SB203580	0.553±0.002	0.001	24.50%
3-MA	0.697±0.278	0.45	4.780%
SB203580	0.728±0.542	0.60	0.5464%

### 2.3 Cobrotoxin对A549细胞集落形成能力影响

Cobrotoxin作用48 h与对照组比较，对A549细胞的集落形成能力有显著抑制作用，不仅集落数目变少，而且体积变小，并随剂量增加抑制能力提高，结果如表 3所示，提示cobrotoxin能抑制A549细胞集落形成。

**3:** cobrotoxin对A549细胞集落形成能力影响 The effect of cobrotoxin on the formation of colony

Cobrotoxin concentration	Colony number	*P* (contrast with control group)	Inhibition rate
Control group	126.5±16.9		
5 μg/mL	98.3±7.4	0.034	22.30%
10 μg/mL	76.0±10.7	0.024	39.50%
20 μg/mL	61.0±12.1	< 0.001	50.50%

### 2.4 Western blot检测各组beclin-1、LC3、pP38及P38蛋白的表达水平

与对照组相比较各浓度的cobrotoxin组beclin-1、pP38蛋白表达明显上调，Ⅱ型/Ⅰ型LC3比值增大，差异有统计学意义，并随cobrotoxin浓度增高，差异越发明显。cobrotoxin+SB203580干预组较cobrotoxin组（10 μg/mL）beclin-1、pP38蛋白表达水平下调，Ⅱ型/Ⅰ型LC3比值减小，差异有统计学意义。P38蛋白表达无明显差异（图 1）。cobrotoxin+3-MA干预组较cobrotoxin组（10 μg/mL）P62蛋白表达升高，beclin-1蛋白表达水平下调，Ⅱ型/Ⅰ型LC3比值减小，差异有统计学意义（图 2）。

**1:**
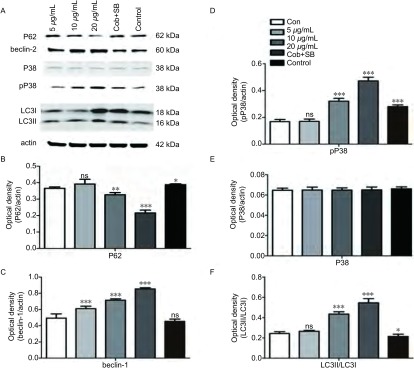
Western blot检测cobrotoxin作用后各相关蛋白表达的情况。A：Western blot法检测不同浓度的crobrotoxin作用A549细胞48 h后各相关蛋白的表达。B、C、D、E、F统计分析各相关蛋白的表达变化。与对照组相比：^*^P < 0.05，^**^P < 0.01，^***^P < 0.001。 The expression of various related protein by Western blot after the intervention of cobrotoxin for 48 h. A549 cells were treated with different concentration of crobrotoxin for 48 h. Protein level was determined by Western blot analysis. A: The expression of related protein after cobrotoxin induction. B, C, D, E, F: Quantification of Western blot analysis. ^*^P < 0.05, ^**^P < 0.01, ^***^P < 0.001, compared with controls.

**2:**
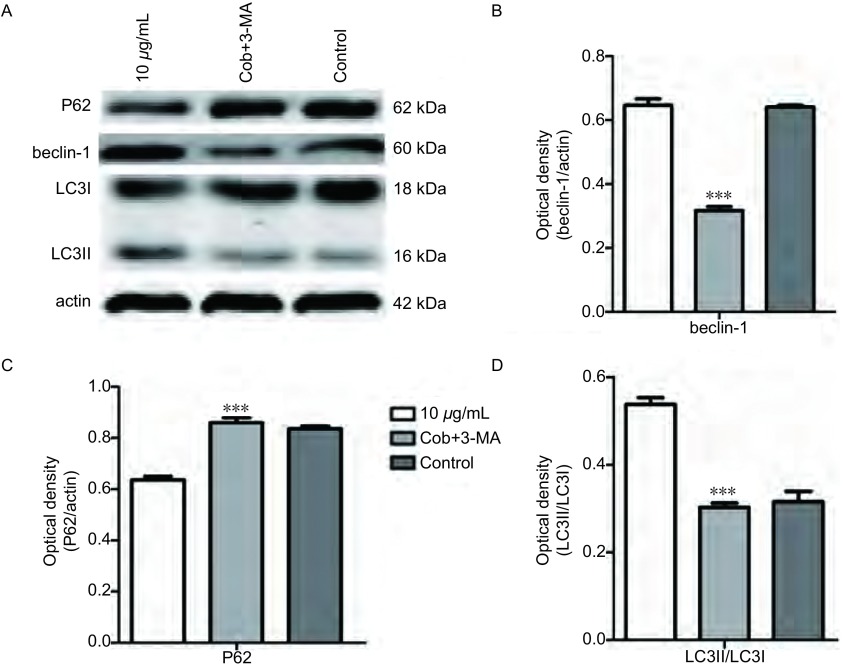
Western blot检测cobrotoxin作用后各相关蛋白表达的情况。A：Western blot法检测10 μg/mL cobrotoxin+5 μmmol/L 3-MA和10 μg/mL cobrotoxin分别作用A549细胞48 h后各相关蛋白的表达。B、C、D：统计分析各相关蛋白的表达变化。与10 μg/mL cobrotoxin组相比，^*^P < 0.05，^**^P < 0.01，^***^P < 0.001。 The expression of various related protein by Western blot after the intervention of 3-MA for 48 h. A549 cells were treated with 10 μg/mL cobrotoxin+5 μmmol/L 3-MA and 10 μg/mL cobrotoxin for 48 h. Protein level was determined by Western blot analysis. A: The expression of related protein after cobrotoxin induction. B, C, D: Quantification of Western blotting analysis. ^*^P < 0.05, ^**^P < 0.01, ^***^P < 0.001, compared with 10 μg/mL cobrotoxin group.

## 3 讨论

Cobrotoxin是眼镜蛇的毒素中分离出来的一种神经毒素，是眼镜蛇毒的主要成分之一，由于镇痛效果确切被美国FDA批准应用于临床。近年来有国内外文献报道cobrotoxin具有抗肿瘤作用，但其具体作用机制尚不明确，推测其抗肿瘤作用途径可能与N型乙酰胆碱受体有关^[6]^。

本实验研究发现cobrotoxin对A549细胞具有明显的抗肿瘤作用，低、中、高三个剂量组抑制率分别为21.8%（5 μg/mL）、34.2%（10 μg/mL）、53.0%（20 μg/mL）。cobrotoxin能有效抑制A549细胞集落形成，抑制率分别为22.3%（5 μg/mL）、39.5%（10 μg/mL）、50.5%（20 μg/mL）。3-MA抑制自噬或SB203580抑制P38-MAPK通路后cobrotoxin对A549细胞的生长抑制作用降低，表明cobrotoxin的抗肿瘤作用与诱导自噬和激活P38-MAPK通路相关。

自噬（autophagy）意为自体吞噬（serf-eating），简称自噬，是溶酶体对细胞内的部分细胞质、细胞器等进行的一系列降解过程的统称。自噬的发生机制尚不明确，受多种机制调节和制约^[7, 8]^。在自噬过程中，LC3Ⅰ转化为LC3Ⅱ并插入新形成的自噬体膜上，因此，LC3通常被用作哺乳动物细胞中自噬的标志蛋白，尤其Ⅱ型/Ⅰ型LC3比值能很好反映自噬的发生^[9, 10]^。自噬基因*Beclin1*是哺乳动物参与自噬的特异性基因，尤其是定位于内质网上的Beclin1是重要的自噬诱导因子，检测其表达可一定程度上反映自噬的发生^[11]^。本实验我们观察到A549细胞经cobrotoxin处理后，Western blot法检测到beclin-1蛋白表达水平、Ⅱ型/Ⅰ型LC3比值上调，P62蛋白表达下调证实了cobrotoxin诱导A549细胞自噬的作用，并随着cobrotoxin浓度增加beclin-1表达水平和Ⅱ型/Ⅰ型LC3比值上调，P62蛋白表达下调。3-MA抑制自噬后，P62蛋白上调，beclin-1表达水平和Ⅱ型/Ⅰ型LC3比值下调，证实了自噬的发生。自噬在肿瘤发生发展中具有双重作用，一方面在血供不足导致营养障碍的情况下，肿瘤细胞通过自噬获得代谢所需能量，维持肿瘤细胞生长，从而促进肿瘤细胞的生长，为保护作用; 另一方面，自噬则导致肿瘤细胞死亡，即第二类程序性细胞死亡或自噬性细胞死亡^[12, 13]^。本实验中与单独使用神经毒素相比联合应用3-MA处理A549细胞其细胞抑制率从34.2%降到27.0%，说明神经毒素所诱导的自噬对A549细胞具有促死亡作用。丝裂原活化蛋白激酶信号转导通路是细胞内重要的信号系统，在将胞外刺激信号转导至细胞及其核内、介导细胞生物学反应（如增殖、分化、转化、凋亡及自噬等）的过程中具有重要的作用^[14-16]^。本实验经cobrotoxin处理后的A549细胞，Western blot法检测到pP38蛋白表达随着cobrotoxin浓度增加而上调。应用P38-MAPK通路特异性抑制剂SB203580后P62蛋白表达上调，beclin-1蛋白表达、Ⅱ型/Ⅰ型LC3比值均下调，据此，我们推断cobrotoxin诱导A549细胞自噬作用可能和激活P38-MAPK信号通路有关。

Cobrotoxin对人肺A549腺癌细胞体外生长有抑制作用，其可能通过活化P38-MAPK通路激活自噬参与抗肿瘤过程。

## References

[1] Rivera MP (2004). Multimodality therapy in the treatment of lung cancer. Semin Respir Crit Care Med.

[2] Wu C, Jiang J, Shi L (2008). Prospective study of chemotherapy incombination with cytokine-induced killer cells in patients suffering from advanced non-small cell lung cancer. Anticancer Res.

[3] Sun P, Xu C, Ren XD (2002). The acute toxicity and tumor inhibition of transplantation tumor of liver cancer by three kinds of venom orally taken. Ji Nan Da Xue Xue Bao (Med Edi).

[4] Ye B, Xie Y, Qin ZH (2011). Antitumor activity of CrTx in human lung adenocarcinoma cell line A549. Acta Pharmacologic Sinica.

[5] Wang JH, He JK, Xie Y (2012). Crotoxin enhances antitumor activity of Gefinitib (Iressa) in human lung squamous carcinoma SK-MES-1 cells. Oncol Rep.

[6] Alama A, Bruzzo C, Cavalieri Z (2011). Inhibition of the nicotinic acetylcholine receptors by cobra venom α-neurotoxins: is there a perspective in lung cancer treatment?. PLoS One.

[7] Tang D, Kang R, Livesey KM (2010). Endogenous HMGBl regulates autophagy. J Cell Biol.

[8] Gewirtz DA (2007). Autophagy as a mechanism of radiation sensitization in breast tumor cells. Autophagy.

[9] Lambert LA, Qiao N, Hunt KK (2008). Autophagy: a novel mechanism of synergistic cytotoxicity between doxorubicin and roscovitine in a sarcoma model. Cancer Res.

[10] Mizushima N, Yoshimori T (2007). How to interpret LC3 immunoblotting. Autophagy.

[11] Cao Y, Klionsky DJ (2007). Physiologic functions of Atg6/Beclin 1: a unique autophagy-related protein. Cell Res.

[12] Sridhar S, Botboly Y, Macian F (2012). Autophagy and disease: always two sides to a problem. J Pathol.

[13] White E, Karp C, Strohecker AM (2010). Role of autophagy in suppression of inflammation and cancer. Curr Opin Cell Biol.

[14] Wu JG, Tang H, Liu ZJ (2011). Angiotensin-(1-7) inhibits vascular remodelling in rat jugular vein grafts via reduced ERK1/2 and p38 MAPK activity. J Int Med Res.

[15] Liu Z, Xu X, Chen L (2012). *Helicobacter pylori* CagA inhibits the expression of Runx3 via Src/MEK/ERK and p38 MAPK pathways in gastric epithelial cell. J Cell Biochem.

[16] Barco Barrantes I, Nebreda AR (2012). Roles of p38 MAPKs in invasion and metastasis. Biochem Soc Trans.

